# Adsorption and caspofungin dosing during continuous renal replacement therapy

**DOI:** 10.1186/s13054-019-2526-2

**Published:** 2019-07-02

**Authors:** Patrick M. Honore, David De Bels, Rachid Attou, Sebastien Redant, Andrea Gallerani, Kianoush Kashani

**Affiliations:** 1 ICU Department, Centre Hospitalier Universitaire Brugmann-Brugmann University Hospital, Place Van Gehuchtenplein,4, 1020 Brussels, Belgium; 2 Division of Nephrology and Hypertension, Division of Pulmonary and Critical Care Medicine, Mayo Clinic, Rochester, USA

In a recent letter by Aguilar et al., the pharmacokinetics of caspofungin during continuous renal replacement therapy (CRRT) and the potential impact of adsorptive membrane capacity on its removal were described [1]. In this study, administered loading caspofungin dose was 50 or 70 mg based on patient weight. As the investigators demonstrated and considering that echinocandins are highly protein-bound and non-renally eliminated, caspofungin removal with CRRT was negligible [1]. Polysulphone membranes that were used by the investigators have minimal adsorptive capacity [2]. However, highly adsorptive membranes (HAMs; e.g., AN69 surface-treated and polymethylmethacrylate filters) could potentially adsorb echinocandins effectively. HAMs’ slow saturation rate would likely increase echinocandins elimination to a clinically relevant level. This, in turn, could lower maximal concentration (Cmax). By reducing Cmax/minimal inhibitory concentration (MIC) ratio for this class of antifungals, their effectiveness could be compromised [3]. This may lead to the need for dose adjustment while on CRRT [4]. In a recent study, Roger et al. showed a higher loading dose (100 mg) is necessary for critically ill patients on CRRT, which is higher than the dosing scheme used by Aguilar et al. [5]. It is essential to highlight that despite the fact that Aguilar and colleagues found minimal caspofungin elimination by CRRT, a third of their patients had subtherapeutic blood levels. Therefore, the standard dosing scheme (50 or 70 mg based on patient weight) when HAMs are used may result in significantly low caspofungin blood levels. We believe, further studies should be conducted focusing on caspofungin dosing on CRRT with HAM filters in order to evaluate the pharmacokinetics of caspofungin and determine its correct dosing strategy.

## Data Availability

Not appropriate.

## Abbreviations

Cmax: maximal concentration
CRRT: continuous renal replacement therapy
HAMs: highly adsorptive membranes
MIC: minimal inhibitory concentration
